# Regulatory T Cells as Immunotherapy

**DOI:** 10.3389/fimmu.2014.00046

**Published:** 2014-02-11

**Authors:** Benjamin D. Singer, Landon S. King, Franco R. D’Alessio

**Affiliations:** ^1^Division of Pulmonary and Critical Care Medicine, Johns Hopkins University, Baltimore, MD, USA

**Keywords:** regulatory T cells, immunotherapeutics, inflammation, tolerance, adoptive transfer, expansion

## Abstract

Regulatory T cells (Tregs) suppress exuberant immune system activation and promote immunologic tolerance. Because Tregs modulate both innate and adaptive immunity, the biomedical community has developed an intense interest in using Tregs for immunotherapy. Conditions that require clinical tolerance to improve outcomes – autoimmune disease, solid organ transplantation, and hematopoietic stem cell transplantation – may benefit from Treg immunotherapy. Investigators have designed *ex vivo* strategies to isolate, preserve, expand, and infuse Tregs. Protocols to manipulate Treg populations *in vivo* have also been considered. Barriers to clinically feasible Treg immunotherapy include Treg stability, off-cell effects, and demonstration of cell preparation purity and potency. Clinical trials involving Treg adoptive transfer to treat graft versus host disease preliminarily demonstrated the safety and efficacy of Treg immunotherapy in humans. Future work will need to confirm the safety of Treg immunotherapy and establish the efficacy of specific Treg subsets for the treatment of immune-mediated disease.

## Introduction

Autoimmunity and alloimmunity protect the host against malignancy and infection; however, unrestrained immune system activation leads to clinical disorders. Induction of immunologic tolerance is essential to improving outcomes in diseases typified by immune system activation: autoimmune disease (1), solid organ transplantation (SOT) (2), and hematopoietic stem cell transplantation (HSCT) (3, 4). In these states, conventional T cells coordinate adaptive immunity and underlie the pathogenesis of autoimmune disease, allograft rejection, and graft versus host disease (GVHD). Current strategies to induce tolerance include immunosuppressive pharmacotherapies that cause functional deletion or anergy of reactive conventional T cells. Toxicity limits use of these drugs, leading investigators to design immunotherapies based on the immune regulatory system. This review focuses on immunotherapy using regulatory T cells (Tregs).

Induction of peripheral immunologic tolerance requires Tregs, which suppress autoimmunity and promote allograft survival (5). Thymic deletion of self-reactive T cells provides a mechanism of *central* tolerance; Tregs represent a *peripheral* system to maintain self-tolerance and prevent over-exuberant immune responses. Mice with mutations in a critical Treg gene (*Foxp3*) develop scurfy, a fatal lymphoproliferative syndrome characterized by multi-organ inflammation (6). IPEX (immunodysregulation, polyendocrinopathy, and enteropathy, X-linked) occurs in humans with loss-of-function *FOXP3* mutations (7). Constitutive expression of the forkhead box protein 3 transcription factor (Foxp3 in mice and FOXP3 in humans) is necessary for Tregs to regulate self-tolerance (8, 9). Polymorphisms of cytotoxic T-lymphocyte antigen 4 (CTLA-4) – a co-signaling molecule with vital importance to Treg function (10) – are also linked to autoimmunity (11). Table 1 lists Treg markers relevant to their use in immunotherapy.

**Table 1:** **Treg markers relevant to their use as immunotherapy with selected references**.

Marker	Alterative name or identifier	Function	Relevance to Treg immunotherapy
Foxp3	Forkhead box protein 3	Transcription factor, master regulator of Treg development and function	Identifies Treg lineage in mice; expressed in human CD4^+^ Tregs (12)
CTLA-4	Cytotoxic T-lymphocyte antigen 4, CD152	Transmits inhibitory signal to APCs	Important mechanism of Treg suppressive function (10)
LAP	Latency-associated peptide	Component of TGF-β latent complex	Identifies Treg subset with TGF-β-mediated function (13)
GITR	Tumor necrosis factor receptor superfamily member 18 (TNFRS18), activation-inducible TNFR family receptor (AITR)	Cell signaling	Important mechanism of Treg suppressive function (14)
ICOS	Inducible T cell costimulator, CD278	Costimulator on T cells	Involved in Treg expansion and IL-10 production, particularly during Th2 inflammation (15)
LAG-3	Lymphocyte activation gene 3, CD223	CD4 homolog with MHC class II binding properties	Expressed on Tregs (16)
CD3	TCR co-receptor complex	TCR signal transduction	Stimulation required for Treg expansion
CD4		Interacts with MHC class II molecules on APCs and amplifies TCR signals	Identifies CD4^+^ lymphocyte subset
CD25	IL-2 receptor α-chain	IL-2 receptor component	Expressed by CD4^+^Foxp3^+^ Tregs but also other T cells (17)
CD28		Costimulator required for T cell activation	Stimulation required for Treg expansion (18)
CD44		Hyaluronic acid receptor	Marker of activated Tregs (19)
CD45RO	Leukocyte common antigen (RO isoform)	Protein tyrosine phosphatase, receptor type, C	Positive Treg marker, also identifies memory T cells
CD45RA	Leukocyte common antigen (RA isoform)	Protein tyrosine phosphatase, receptor type, C	Minor Treg marker, also identifies naïve T cells
CD49b	Integrin VLA-4 α4β1 α-chain	Cell adhesion and signaling	Expressed on Tregs (16)
CD62L	L-selectin	Lymphocyte cell adhesion molecule	May be marker of effective disease-modulating Treg subset (20, 21)
CD69	Transmembrane C-Type lectin	Cell signaling	Marker of activated Tregs that suppress via membrane-bound TGF-β1 (22)
CD127	IL-7 receptor α-chain	IL-7 receptor	Negative Treg marker (23)

Immunologically, Tregs comprise a subset of CD4^+^ lymphocytes that suppresses activation, proliferation, and effector responses of both innate and adaptive immune cells (17). Functional Tregs also express the interleukin-2 (IL-2) receptor α-chain (CD25), although activated conventional T cells also transiently express CD25. Like conventional T cells, Tregs require T cell receptor (TCR) stimulation and costimulation for activation. *Natural Tregs* (nTregs) are derived centrally in the thymus (12); *induced Tregs* (iTregs) upregulate FOXP3 in the periphery following antigen exposure and, for example, stimulation from transforming growth factor β (TGF-β) (24). nTregs comprise 5–10% of the circulating CD4^+^ population. Circulating and tissue iTreg numbers depend on anatomic location as well as specific inflammatory environmental conditions. Abbas et al. recently published recommendations for Treg nomenclature (25); in this review, we will use nomenclature used by cited authors.

Gershon proposed using Tregs for immunotherapy decades ago (26); however, clinical implementation of protocols employing Treg immunotherapy has proved challenging. In this review, we discuss strategies for using Tregs as immunotherapy, address barriers to the use of Tregs, provide promising examples of Treg immunotherapy in animal models and clinical trials, and conclude with future directions for the field.

## Practical Use of Tregs for Immunotherapy

**Figure 1:**
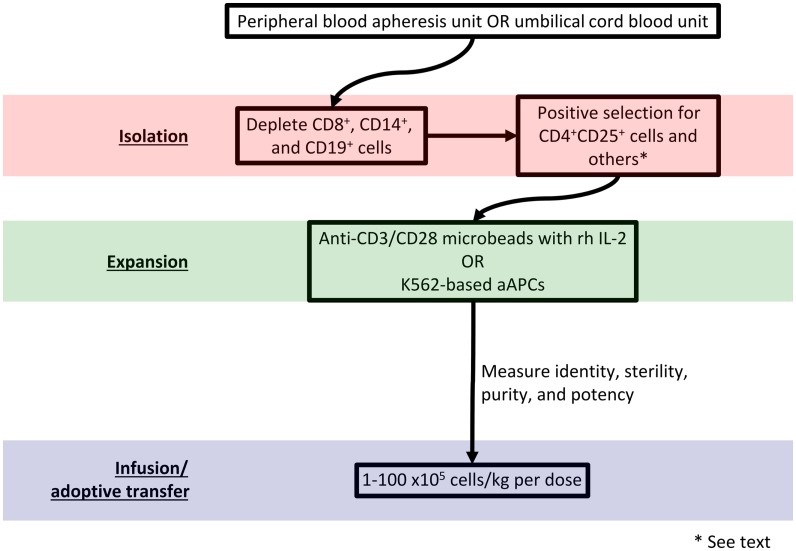
**Schematic of a strategy to isolate, expand, and infuse Tregs**.

Adoptive transfer of autologous or donor-derived Tregs represents an exciting immunotherapeutic strategy (27). Broadly, protocols for adoptive transfer call for Treg isolation from the host or a donor, enrichment, expansion, and re-infusion. Figure 1 diagrams such a protocol. Advantages of an *ex vivo* expansion strategy include the ability to perform careful cellular phenotyping and govern the dose of administered cells (28). As the contribution of reduced Treg *number* versus reduced Treg *function* remains unclear in autoimmune pathogenesis (29, 30), it is advantageous from an experimental perspective to maintain control over the phenotype and number of infused Tregs.

Peripheral or banked umbilical cord blood (UCB) may serve as a Treg source. A frozen UCB unit yields approximately 5–7.5 × 10^6^ Tregs; an adult peripheral blood apheresis unit can yield on the order of 10^8^ Tregs (28). Successful isolation requires labeling cell surface markers with a tagged antibody and sorting via fluorescence-activated cell sorting (FACS) or magnetic bead separation. Unfortunately, no cell surface markers uniquely identify Tregs. Although Foxp3 expression specifies the Treg lineage in mice (31), T cells promiscuously express FOXP3 in humans (32). Regardless, FOXP3 detection requires cell permeabilization, which renders cells unusable for adoptive transfer. Because activated CD4^+^ conventional T cells may also transiently express CD25, patterns of CD127 (the IL-7 receptor α-chain) (23), CD49b (the integrin VLA-4 α4β1 α-chain) (16), lymphocyte activation gene 3 (LAG-3) (16), CD45RA, CD45RO, and latency-associated peptide (LAP) (13) can identify Tregs and facilitate their isolation. Although Tregs express CTLA-4, glucocorticoid-induced TNFR family related gene (GITR) (14), CD69 (22), and CD44 (19), activated non-Tregs may also express these markers.

*Ex vivo* stimulation with anti-CD3/CD28 microbeads in the presence of recombinant human (rh) IL-2 expands Tregs for subsequent manipulation (33, 34). The resultant Tregs have polyclonal reactivity due to non-specific TCR stimulation. However, other protocols generate donor alloantigen-specific Tregs for establishment of allograft tolerance. In one method, Tregs are expanded in the presence of donor antigen-presenting cells (APCs). These Tregs have more potency than polyclonally reactive Tregs and demonstrate a more favorable safety profile *in vivo* (35, 36). Retroviral vector transduction of genes encoding TCRs with known antigen specificities also produces alloantigen-reactive Tregs (37). Anti-CD3 antibody-loaded K562-based artificial antigen-presenting cells (aAPCs) may efficiently expand Tregs with a high level of purity and potency (38, 39). Genetic modification that adds cell surface molecules and secreted factors to K562-based aAPCs could further refine the expanded Treg population (40).

It remains unclear what constitutes a therapeutic dose of Tregs. The therapeutic dose in a given application will depend on Treg potency, disease state and activity, and whether protocols employ polyclonal or antigen-specific Tregs (41). In a phase I dose-escalation trial of Tregs for prevention of acute GVHD, Blazar’s group used Treg dosages between 1 × 10^5^ and 30 × 10^5^/kg (42). Di Ianni et al. used 40 × 10^5^/kg of Treg in a similar trial (43). Based on animal studies, effective immunosuppression and tolerance induction may require up to 1 × 10^9^ Tregs per infusion (44). To that end, Hoffmann et al. reported a protocol capable of a 4 × 10^4^-fold Treg expansion in 3–4 weeks (45); however, the purity and phenotype of these cells was difficult to ascertain. Using the aAPC method, a 1 × 10^3^-fold expansion of human peripheral blood Tregs can be performed in approximately 3 weeks (39).

*Ex vivo* conversion of CD4^+^CD25^−^ naïve T cells into iTregs with suppressor function represents an alternative strategy to *ex vivo* nTreg isolation and expansion (46). Exposure of naïve CD4^+^CD25^−^ or CD4^+^CD45RO^−^ T cells to TGF-β (47) with the addition of IL-2, IL-10, or vitamin D3 (48), indoleamine 2,3-dioxygenase (49), all-trans retinoic acid (50), *Foxp3*-expressing retroviruses (12), or epigenetic modifiers (DNA methyltransferase inhibitors or histone deacetylase inhibitors) (51) accomplishes such a conversion. Lan et al. have suggested that iTregs have more potency than nTregs on a cell-by-cell basis (52), making strategies that expand iTregs attractive for Treg immunotherapy. Future work will need to validate methods of identifying nTregs versus iTregs in humans and assessing their stability and plasticity (53).

A variety of strategies induce Treg number or potency *in vivo* including expansion of nTregs and conversion of non-Tregs to iTregs (54). For example, treating mice prior to allografting with a donor alloantigen and a non-depleting anti-CD4 antibody achieves Treg expansion. Tregs generated by this method prevent allograft rejection (55–58). Moreover, adoptive transfer of Tregs isolated from treated animals abrogates rejection (59). nTregs isolated from naïve animals may also prevent rejection, although long-term allograft survival requires 10-fold more Tregs compared with Tregs isolated from tolerant mice treated with antigen exposure alone (60).

Injection of IL-2/IL-2 monoclonal antibody (mAb) complexes into mice results in a 10-fold *in vivo* Treg expansion (61). Mice treated with this protocol display immunologic tolerance and resistance to experimental autoimmune encephalomyelitis and islet allograft rejection. Simultaneous injection of IL-2/IL-2 mAb complexes and recombinant granulocyte-colony stimulating factor (G-CSF), which causes expansion of myeloid-derived suppressor cells (MDSCs), augments induction of immunologic tolerance. Expansion of MDSCs in addition to Tregs supports MHC class II-mismatched skin allograft survival (62). In a phase 1 dose-escalation trial of subcutaneous IL-2 to treat active chronic GVHD, daily low-dose IL-2 was well-tolerated and led to sustained Treg expansion with improvement in GVHD manifestations (63).

Other pharmacotherapies target particular facets of Treg biology. IL-2-dependent STAT-5 activates Tregs (64), whereas effector T cells employ the phosphoinositide 3-kinase/Akt/mTOR pathway (65). The mTOR inhibitor rapamycin exploits the latter pathway to preferentially expand Tregs (66–68). Clinically, rapamycin increases the number of CD62L^high^ Tregs in the peripheral blood of lung transplant recipients (20) and expands the Treg population in renal transplant patients (69). Anti-thymocyte globulin (ATG), a T cell-depleting polyclonal antibody that promotes Treg generation in mice (70), supports allograft survival when combined with CTLA-4-Ig and rapamycin in a MHC-mismatched skin allograft model (71). In that model, memory T cell-Treg balance shifted in favor of Tregs. Glucocorticoids have broad effects on T cells; however, glucocorticoids may interact with Langerhans cells to promote Treg expansion in contact dermatitis (72). The lymphocyte depleting mAb alemtuzumab (anti-CD52 mAb) may have favorable effects on Treg survival when combined with rapamycin (73). Standard dosages of calcineurin inhibitors such as cyclosporine A and tacrolimus impair Tregs (74). However, treatment with low-dose cyclosporine may increase Treg numbers in the skin of atopic dermatitis patients (75). Compared to conventional doses, low doses of calcineurin inhibitors may allow patients to continue the production of IL-2, which Tregs require for expansion and survival (76, 77). Weng et al. published that the proteasome inhibitor bortezomib reduced acute GVHD severity and prolonged survival time by triggering generation of Tregs (78). A recently reported high-throughput screening assay may increase the number of known compounds with positive effects on Tregs (79).

## Barriers to Use of Tregs for Immunotherapy

Regulatory T cell functional stability represents a challenge for using Tregs for immunotherapy. A minor population of Foxp3^+^ cells loses Foxp3 expression over time; these “ex-Foxp3” cells may display an activated conventional T cell phenotype and become pathogenic *in vivo* (80). Loss of Foxp3 expression has been associated with a pro-inflammatory microenvironment and switching to an effector T cell phenotype characterized by IL-17 and interferon-γ secretion (81–83). While Tregs delivered to a normal host tend to retain their suppressive function, a proportion of Tregs adoptively transferred into a lymphopenic environment may differentiate into pathogenic T cells (84, 85). Exploiting the epigenetic control of the *Foxp3* gene could maintain Foxp3 expression and Treg stability (85–87). Both DNA methylation at the *Foxp3* upstream control regions (88) and chromatin remodeling (89) help determine Treg plasticity. Pharmacologic DNA methyltransferase inhibitors or histone deacetylase inhibitors could maintain Treg fidelity following adoptive transfer (51). IL-2 therapy might also promote Treg stability after infusion (63).

Despite the fact that some costimulatory pathways differentially affect conventional T cells versus Tregs, no single pathway completely selects for a specific T cell subset (90). Therefore, administration of pharmaceuticals that stimulate Tregs may also activate conventional T cells (off-cell effect). Indeed, a phase I clinical trial of TGN1412 – a super-agonistic anti-CD28 antibody – caused massive cytokine storm and multi-organ dysfunction in six healthy adults who required intensive care following administration of the drug (91). The misadventure with TGN1412 highlights the risks of drugs designed to modulate T cell activity without selectively targeting specific T cell subsets. As above, drugs that modify T cell epigenetic signatures may add specificity to T cell pharmacotherapy (92–94).

Memory T cells provide a significant barrier to the induction of clinical tolerance (95), and depleting donor-reactive T cells permits Tregs to control allograft rejection (96). Therefore, investigators desire drug protocols that functionally deplete memory T cells while maintaining immunoregulation. Alefacept, an LFA-3-Ig fusion protein that polymerizes CD2, leads to selective memory T cell elimination. When administered with CTLA-4-Ig, alefacept prevents acute rejection and promotes kidney transplant engraftment in a non-human primate model (97). Efalizumab, an anti-LFA-1 antibody, showed efficacy in islet-cell transplantation (98) but was withdrawn from the market after four patients with psoriasis developed progressive multifocal leukoencephalopathy (99). Functional Tregs themselves also potently suppress memory T cell proliferation in humans and may not require additional pharmacotherapy to overcome the effect of memory T cells if administered in sufficient dosages (100).

The United States Food and Drug Administration mandates documentation of sterility, identity, purity, and potency of a cell therapy product before administration to patients (21 CFR §1271). Sterility and identity are relatively facile to demonstrate; purity and potency are more problematic. Investigators will need to empirically determine the acceptable level of non-Treg contamination in cell preparations. CpG demethylation of the *Foxp3* conserved non-coding sequence 2 (CNS2) identifies committed suppressive Tregs (85, 86); therefore, methylation status of the *FOXP3* CNS2 region may indicate Treg purity and stability in cells destined for clinical use. As Tregs have many mechanisms of action, difficulty exists in elucidating which mechanisms regulate a specific disease in an inflammatory environment (101, 102). Therefore, *in vitro* assays – such as the ability of Tregs to inhibit conventional T cell proliferation – may inadequately describe the potency of cell preparations. For example, Golovina et al. reported that CD4^+^ T cells expanded in the presence of rapamycin were effective in an *in vitro* suppression assay, but these cells failed to function in an *in vivo* xeno-GVHD model (18). These findings imply that investigators may need to develop disease-specific Treg potency testing systems prior to use in humans. Non-human primates have been used to validate SOT protocols (103), but even these models may lead to erroneous conclusions (104).

Cryopreservation of Treg cell preparations presents technical challenges (105), although investigators have developed feasible cryopreservation protocols. One popular method involves liquid nitrogen cryopreservation with 20% human pooled serum and 15% DMSO. After 1 year, Tregs showed 70–80% viability; stimulation and subsequent expansion restored Treg function to pre-cryopreservation levels (106). Strategies to freeze already-expanded Tregs also exist. In their seminal clinical trial of Treg immunotherapy for GVHD (reviewed below), Brunstein et al. cryopreserved *ex vivo*-expanded Tregs that were not initially infused (42). Their protocol used a freezing medium containing Plasma-Lyte A™ (Baxter, Deerfield, IL, USA), 10% DMSO, and human serum albumin. The thawed cells had an immediate post-thaw viability exceeding 50%. However, an increase in peripheral blood Tregs following infusion was not observed, whereas the authors had observed a significant increase following the first infusion of non-cryopreserved Tregs. Other authors using a mouse GVDH model detected preserved *in vivo* suppressive function after thawing aAPC-expanded Tregs (39). Further refinement of cryopreservation strategies could facilitate an “on demand” treatment for acute inflammatory disease or acute allograft rejection without the time delay required for *ex vivo* isolation and expansion.

Potential adverse effects of Treg infusion or expansion include those associated with immunosuppression, including infection and malignancy. Interestingly, Di Ianni et al. observed improved immunity to opportunistic pathogens in their trial of Treg infusion for GVHD prevention following HSCT (43). Brunstein et al. similarly reported no increased risk of infection following Treg infusion for acute GVHD (42). Numerous studies implicate Tregs in suppressing anti-tumor immunity [reviewed in (107)]. Future study will need to carefully examine the effect of Treg manipulation on infectious risk and neoplasia.

## Examples of Treg Immunotherapy

### Allograft tolerance

Graft versus host disease results from donor T cell-mediated systemic inflammation that overwhelms immune regulatory mechanisms following allogeneic HSCT (108). Clinical disease results when donor (i.e., graft) cells recognize host cells as foreign and incite an inflammatory reaction. Inflammation often causes tissue damage despite routine post-HSCT immunosuppressive pharmacotherapy designed to dampen T cell alloreactivity. In contrast to SOT, HSCT eventually fosters the development of tolerance, as donor APCs and T cells replace host leukocytes. Therefore, risk of alloreactive immunity peaks in the first few months following HSCT, highlighting GVHD as an ideal application for Treg immunotherapy. The availability of Tregs from HSCT donors makes Treg immunotherapy protocols particularly feasible.

Strong pre-clinical work supports the use of CD4^+^CD25^+^ Tregs to suppress acute GVHD (109). Trzonkowski et al. reported the first two cases of *ex vivo*-expanded donor-derived Tregs to successfully treat post-HSCT GVHD (33). A phase I dose-escalation trial demonstrated the safety profile and efficacy of human UCB-derived partially HLA-matched *ex vivo*-expanded Tregs in reducing the incidence of grades II–IV GVHD in 23 patients compared with 108 controls (42). These investigators isolated Tregs with anti-CD25 magnetic beads, expanded them with anti-CD3/CD28 microbeads and rh IL-2, and infused the expanded Tregs at the time of HSCT. Di Ianni et al. used adult expanded Tregs isolated from the same HLA-haploidentical donor to assess safety and efficacy in prevention of chronic GVHD in 28 patients undergoing HLA-haploidentical HSCT for high-risk acute leukemia (43). These patients also received donor conventional T cells to enhance immune reconstitution and to promote the graft versus leukemia effect. Chronic GVHD developed in only 2 of 28 patients. Other trials of Treg adoptive transfer are ongoing (110, 111).

In 1995, Sakaguchi et al. published their watershed observation that Tregs from naïve mice prevented rejection of allogeneic skin grafts in nude mice given CD25^−^ T cells (17). Their work laid the foundation for the use of Treg immunotherapy to promote tolerance following SOT. Indeed, induction of tolerance to alloantigen via costimulatory blockade requires Tregs (112). In a MHC-mismatched mouse orthotopic lung transplant model, blockade of CD154 increased Tregs and was associated with attenuation of acute cellular rejection (113). In a chimeric humanized mouse system, *ex vivo*-expanded Tregs prevented transplant arteriosclerosis *in vivo* by limiting effector cell function and allograft infiltration (114). Clinical trials of Treg adoptive transfer to promote SOT tolerance have not been published; however, this review highlights pre-clinical work that could inform the design of post-SOT Treg immunotherapy protocols. Results from The ONE Study (115) should shed light on Treg immunotherapy for induction of tolerance following SOT.

### Atopic disease

Atopy is a complex immune phenomenon characterized by Th2-predominant inflammation, production of allergen-specific immunoglobulin E (IgE), attraction of pro-inflammatory cells, and the degranulation of effector cells (e.g., mast cells) (116). Literature supports a functional role for Tregs in maintaining allergen tolerance in normal individuals. Indeed, an imbalance between Tregs and Th2 cells leads to an atopic phenotype (117). The E3 ligase Itch has recently been identified as a critical protein controlling the Treg response to Th2 inflammation (118) and may be a therapeutic target in atopic disease states. Allergen-specific immunotherapy decreases allergen-specific T-cell proliferation, Th2-type cytokine production, and inflammatory cell activity (119). Generation of IL-10-producing Tregs may be a prominent mechanism underlying these findings (15). The antidepressant drug desipramine appears to alleviate allergic rhinitis by regulating Tregs and Th17 cells (120). Although clinical trials have not attempted adoptive transfer of Tregs for allergic disease, protocols to expand allergen-specific Tregs may potentially benefit atopic patients.

### Autoimmune disease

Numerous studies have demonstrated diminished numbers of peripheral blood Tregs in patients with autoimmune conditions and that a Treg deficit associates with disease development (121). Redistribution of the Treg population to the tissue compartment does not fully explain the association between peripheral blood Treg deficiency and disease development (122). Moreover, some autoimmune conditions alter the functional activity of Tregs. Such a functional alteration exists in rheumatoid arthritis (123) and multiple sclerosis (124).

Failure to control islet-specific conventional T cells results in type 1 diabetes mellitus (DM1). Risk of DM1 increases with the loss of FOXP3-expressing Tregs (125), and Treg adoptive transfer to non-obese diabetic (NOD) mice can prevent the development of DM1 (41, 126). Interestingly, 80% of IPEX patients develop DM1 in infancy (127). Marek-Trzonkowska et al. recently published a study demonstrating that a donor-derived CD4^+^CD25^high^CD127^−^Treg infusion preserves β-cell function and may delay DM1 onset in children (128).

Despite positive results in DM1 and other animal models of autoimmune disease including myasthenia gravis (129), adoptive transfer of nTregs has not met with universal success. Adoptive transfer of nTregs had only a nominal effect on controlling disease progression in a collagen-induced arthritis model (130) and failed to suppress glomerulonephritis and sialadenitis in mice with established lupus (131). nTregs have had variable achievement in controlling other Th17-mediated autoimmune diseases (132). The inability of nTregs to treat many autoimmune disorders may relate to pro-inflammatory cytokines that suppress their function (123, 133) or convert them to pathogenic T cells upon adoptive transfer. Additionally, activated Th17 cells may resist many suppressive mechanisms employed by nTregs. iTregs might be a more appropriate Treg subset for use in autoimmune immunotherapy, as data suggest that iTregs more effectively suppress autoimmune activation compared with nTregs possibly due to differential stability in inflammatory environments (52).

### Acute inflammatory disease

Our group established that resolution of experimental murine acute lung injury requires Tregs (134). Mice lacking all mature lymphocytes (*Rag-1*^−/−^) do not resolve their injury by day 10 following an intratracheal injection of *E. coli* lipopolysaccharide (LPS), whereas wild-type mice normalize. Adoptive transfer of 1 × 10^6^ congenic CD4^+^CD25^+^ cells up to 48 h after receiving LPS restores resolution in *Rag-1*^−/−^ mice to that of wild-type mice. Moreover, Treg adoptive transfer limits fibroproliferation following acute lung inflammation (135). Tregs also promote repair from ischemic acute kidney injury (136) and have protective immunomodulatory effects following acute stroke (137). These findings not only demonstrate the importance of Tregs in tissue injury repair but also open the door to studying Treg immunotherapy for other acute inflammatory conditions.

## Future Directions and Conclusion

Most clinical trials of Treg immunotherapy employed adoptive transfer of CD4^+^CD25^+^ or CD4^+^CD25^+^CD127^−^cells. However, more precisely defined human Treg subsets exist; exploiting these Treg subsets may benefit certain disease states. For example, inducible costimulator-expressing (ICOS^+^) Tregs secrete more IL-10 than ICOS^−^ Tregs and could improve conditions characterized by relative IL-10 deficiency, such as atopic disease (15). ICOS^+^ Tregs may also play an important role in dendritic cell function (138). Another example is the CD62L^+^ subpopulation of CD4^+^CD25^+^ Tregs, which appears to most effectively treat acute GVHD (21). In rheumatoid arthritis, abnormal Treg function may stem from defective CTLA-4 (139); therefore, augmentation of functional CTLA-4^+^ Tregs may be advantageous in rheumatoid arthritis. Understanding Treg subset trafficking and survival via chemokine and integrin signals will be key to selecting appropriate Treg subsets for a given application (140).

Genetic reprograming of Tregs, possibly using clinical-grade lentiviral vectors, represents an attractive strategy to fine tune Treg subpopulations (141). Induction of a chimeric immune receptor into Tregs prevented mouse models of experimental autoimmune encephalomyelitis (142) and colitis (143). Engineered TCRs that redirect Treg specificity could also improve Treg potency (144), as Varela-Rohena et al. demonstrated in conventional T cells (145).

Use of Tregs for immunotherapy has a solid pre-clinical database, and emerging data support the safety and efficacy of Treg immunotherapy protocols in patients whose clinical scenario requires induction of clinical tolerance. Both *ex vivo* expansion with adoptive transfer and *in vivo* manipulation to expand and augment the function of endogenous Tregs represent promising strategies to treat autoimmune and alloimmune conditions. In order for clinically feasible Treg immunotherapy protocols to succeed, investigators will need to surmount significant barriers including Treg stability, isolation and storage of Treg subpopulations, and off-target effects of *in vivo* Treg strategies. Because immune dysregulation underlies myriad clinical disorders, designing safe and effective immunotherapies that utilize Tregs could be of great benefit.

## Author Contributions

Benjamin D. Singer and Franco R. D’Alessio made substantial contributions to the conception of the work, drafting the work, and critically revising it for important intellectual content. Landon S. King provided substantial contributions to the design of the work, and has critically revised the manuscript. All authors share final approval of the version to be published. All authors agree to be accountable for all aspects of the work in ensuring that questions related to the accuracy or integrity of any part of the work are appropriately investigated and resolved.

## Conflict of Interest Statement

The authors declare that the research was conducted in the absence of any commercial or financial relationships that could be construed as a potential conflict of interest.

## References

[1] Christen U von Herrath MG Initiation of autoimmunity. Curr Opin Immunol (2004) 16:759–67 10.1016/j.coi.2004.09.002 15511670

[2] Rosenberg AS Singer A Cellular basis of skin allograft rejection: an in vivo model of immune-mediated tissue destruction. Annu Rev Immunol (1992) 10:333–58 10.1146/annurev.iy.10.040192.002001 1590990

[3] Trenado A Charlotte F Fisson S Yagello M Klatzmann D Salomon BL Recipient-type specific CD4+CD25+ regulatory T cells favor immune reconstitution and control graft-versus-host disease while maintaining graft-versus-leukemia. J Clin Invest (2003) 112:1688–96 10.1172/JCI17702 14660744 PMC281639

[4] Edinger M Hoffmann P Ermann J Drago K Fathman CG Strober S CD4+CD25+ regulatory T cells preserve graft-versus-tumor activity while inhibiting graft-versus-host disease after bone marrow transplantation. Nat Med (2003) 9:1144–50 10.1038/nm915 12925844

[5] Sakaguchi S Sakaguchi N Shimizu J Yamazaki S Sakihama T Itoh M Immunologic tolerance maintained by CD25+ CD4+ regulatory T cells: their common role in controlling autoimmunity, tumor immunity, and transplantation tolerance. Immunol Rev (2001) 182:18–32 10.1034/j.1600-065X.2001.1820102.x 11722621

[6] Brunkow ME Jeffery EW Hjerrild KA Paeper B Clark LB Yasayko SA Disruption of a new forkhead/winged-helix protein, scurfin, results in the fatal lymphoproliferative disorder of the scurfy mouse. Nat Genet (2001) 27:68–73 10.1038/83784 11138001

[7] Bennett CL Christie J Ramsdell F Brunkow ME Ferguson PJ Whitesell L The immune dysregulation, polyendocrinopathy, enteropathy, X-linked syndrome (IPEX) is caused by mutations of FOXP3. Nat Genet (2001) 27:20–1 10.1038/83713 11137993

[8] Fontenot JD Gavin MA Rudensky AY Foxp3 programs the development and function of CD4+CD25+ regulatory T cells. Nat Immunol (2003) 4:330–6 10.1038/ni904 12612578

[9] Josefowicz SZ Rudensky A Control of regulatory T cell lineage commitment and maintenance. Immunity (2009) 30:616–25 10.1016/j.immuni.2009.04.009 19464984 PMC4410181

[10] Wing K Onishi Y Prieto-Martin P Yamaguchi T Miyara M Fehervari Z CTLA-4 control over Foxp3+ regulatory T cell function. Science (2008) 322:271–5 10.1126/science.1160062 18845758

[11] Scalapino KJ Daikh DI CTLA-4: a key regulatory point in the control of autoimmune disease. Immunol Rev (2008) 223:143–55 10.1111/j.1600-065X.2008.00639.x 18613834

[12] Hori S Nomura T Sakaguchi S Control of regulatory T cell development by the transcription factor Foxp3. Science (2003) 299:1057–61 10.1126/science.1079490 12522256

[13] Gandhi R Farez MF Wang Y Kozoriz D Quintana FJ Weiner HL Cutting edge: human latency-associated peptide+ T cells: a novel regulatory T cell subset. J Immunol (2010) 184:4620–4 10.4049/jimmunol.0903329 20368276 PMC2904991

[14] Levings MK Sangregorio R Sartirana C Moschin AL Battaglia M Orban PC Human CD25+CD4+ T suppressor cell clones produce transforming growth factor beta, but not interleukin 10, and are distinct from type 1 T regulatory cells. J Exp Med (2002) 196:1335–46 10.1084/jem.20021139 12438424 PMC2193983

[15] Bohle B Kinaciyan T Gerstmayr M Radakovics A Jahn-Schmid B Ebner C Sublingual immunotherapy induces IL-10-producing T regulatory cells, allergen-specific T-cell tolerance, and immune deviation. J Allergy Clin Immunol (2007) 120:707–13 10.1016/j.jaci.2007.06.013 17681368

[16] Gagliani N Magnani CF Huber S Gianolini ME Pala M Licona-Limon P Coexpression of CD49b and LAG-3 identifies human and mouse T regulatory type 1 cells. Nat Med (2013) 19:739–46 10.1038/nm.3179 23624599

[17] Sakaguchi S Sakaguchi N Asano M Itoh M Toda M Immunologic self-tolerance maintained by activated T cells expressing IL-2 receptor alpha-chains (CD25). Breakdown of a single mechanism of self-tolerance causes various autoimmune diseases. J Immunol (1995) 155:1151–64 7636184

[18] Golovina TN Mikheeva T Suhoski MM Aqui NA Tai VC Shan X CD28 costimulation is essential for human T regulatory expansion and function. J Immunol (2008) 181:2855–68 18684977 10.4049/jimmunol.181.4.2855 PMC2556987

[19] Bollyky PL Falk BA Long SA Preisinger A Braun KR Wu RP CD44 costimulation promotes FoxP3+ regulatory T cell persistence and function via production of IL-2, IL-10, and TGF-beta. J Immunol (2009) 183:2232–41 10.4049/jimmunol.0900191 19635906 PMC3057032

[20] Lange CM Tran TYV Farnik H Jungblut S Born T Wagner TO Increased frequency of regulatory T cells and selection of highly potent CD62L+ cells during treatment of human lung transplant recipients with rapamycin. Transpl Int (2010) 23:266–76 10.1111/j.1432-2277.2009.00973.x 19804585

[21] Ermann J Hoffmann P Edinger M Dutt S Blankenberg FG Higgins JP Only the CD62L+ subpopulation of CD4+CD25+ regulatory T cells protects from lethal acute GVHD. Blood (2005) 105:2220–6 10.1182/blood-2004-05-2044 15546950

[22] Han Y Guo Q Zhang M Chen Z Cao X CD69+ CD4+ CD25- T cells, a new subset of regulatory T cells, suppress T cell proliferation through membrane-bound TGF-beta 1. J Immunol (2009) 182:111–20 19109141 10.4049/jimmunol.182.1.111

[23] Seddiki N Santner-Nanan B Martinson J Zaunders J Sasson S Landay A Expression of interleukin (IL)-2 and IL-7 receptors discriminates between human regulatory and activated T cells. J Exp Med (2006) 203:1693–700 10.1084/jem.20060468 16818676 PMC2118333

[24] Karim M Kingsley CI Bushell AR Sawitzki BS Wood KJ Alloantigen-induced CD25+CD4+ regulatory T cells can develop in vivo from CD25-CD4+ precursors in a thymus-independent process. J Immunol (2004) 172:923–8 14707064 10.4049/jimmunol.172.2.923

[25] Abbas AK Benoist C Bluestone JA Campbell DJ Ghosh S Hori S Regulatory T cells: recommendations to simplify the nomenclature. Nat Immunol (2013) 14:307–8 10.1038/ni.2554 23507634

[26] Gershon RK A disquisition on suppressor T cells. Transplant Rev (1975) 26:170–85 1101469 10.1111/j.1600-065x.1975.tb00179.x

[27] June CH Blazar BR Clinical application of expanded CD4+25+ cells. Semin Immunol (2006) 18:78–88 10.1016/j.smim.2006.01.006 16458015

[28] Riley JL June CH Blazar BR Human T regulatory cell therapy: take a billion or so and call me in the morning. Immunity (2009) 30:656–65 10.1016/j.immuni.2009.04.006 19464988 PMC2742482

[29] Kukreja A Cost G Marker J Zhang C Sun Z Lin-Su K Multiple immuno-regulatory defects in type-1 diabetes. J Clin Invest (2002) 109:131–40 10.1172/JCI13605 11781358 PMC150819

[30] Lindley S Dayan CM Bishop A Roep BO Peakman M Tree TIM Defective suppressor function in CD4(+)CD25(+) T-cells from patients with type 1 diabetes. Diabetes (2005) 54:92–9 10.2337/diabetes.54.1.92 15616015

[31] Fontenot JD Rasmussen JP Williams LM Dooley JL Farr AG Rudensky AY Regulatory T cell lineage specification by the forkhead transcription factor foxp3. Immunity (2005) 22:329–41 10.1016/j.immuni.2005.01.016 15780990

[32] Allan SE Crome SQ Crellin NK Passerini L Steiner TS Bacchetta R Activation-induced FOXP3 in human T effector cells does not suppress proliferation or cytokine production. Int Immunol (2007) 19:345–54 10.1093/intimm/dxm014 17329235

[33] Trzonkowski P Bieniaszewska M Juscinska J Dobyszuk A Krzystyniak A Marek N First-in-man clinical results of the treatment of patients with graft versus host disease with human ex vivo expanded CD4+CD25+CD127- T regulatory cells. Clin Immunol (2009) 133:22–6 10.1016/j.clim.2009.06.001 19559653

[34] Earle KE Tang Q Zhou X Liu W Zhu S Bonyhadi ML In vitro expanded human CD4+CD25+ regulatory T cells suppress effector T cell proliferation. Clin Immunol (2005) 115:3–9 10.1016/j.clim.2005.02.017 15870014 PMC7128890

[35] Golshayan D Jiang S Tsang J Garin MI Mottet C Lechler RI In vitro-expanded donor alloantigen-specific CD4+CD25+ regulatory T cells promote experimental transplantation tolerance. Blood (2007) 109:827–35 10.1182/blood-2006-05-025460 17003369

[36] Sagoo P Ali N Garg G Nestle FO Lechler RI Lombardi G Human regulatory T cells with alloantigen specificity are more potent inhibitors of alloimmune skin graft damage than polyclonal regulatory T cells. Sci Transl Med (2011) 3:83ra42 10.1126/scitranslmed.3002076 21593402 PMC3776382

[37] Jiang S Tsang J Game DS Stevenson S Lombardi G Lechler RI Generation and expansion of human CD4+ CD25+ regulatory T cells with indirect allospecificity: potential reagents to promote donor-specific transplantation tolerance. Transplantation (2006) 82:1738–43 10.1097/01.tp.0000244932.29542.9e 17198269

[38] Hippen KL Harker-Murray P Porter SB Merkel SC Londer A Taylor DK Umbilical cord blood regulatory T-cell expansion and functional effects of tumor necrosis factor receptor family members OX40 and 4-1BB expressed on artificial antigen-presenting cells. Blood (2008) 112:2847–57 10.1182/blood-2008-01-132951 18645038 PMC2556620

[39] Hippen KL Merkel SC Schirm DK Sieben CM Sumstad D Kadidlo DM Massive ex vivo expansion of human natural regulatory T cells (T(regs)) with minimal loss of in vivo functional activity. Sci Transl Med (2011) 3:83ra41 10.1126/scitranslmed.3001809 21593401 PMC3551476

[40] Suhoski MM Golovina TN Aqui NA Tai VC Varela-Rohena A Milone MC Engineering artificial antigen-presenting cells to express a diverse array of co-stimulatory molecules. Mol Ther (2007) 15:981–8 10.1038/mt.sj.6300134 17375070 PMC3932757

[41] Tang Q Henriksen KJ Bi M Finger EB Szot G Ye J In vitro-expanded antigen-specific regulatory T cells suppress autoimmune diabetes. J Exp Med (2004) 199:1455–65 10.1084/jem.20040139 15184499 PMC2211775

[42] Brunstein CG Miller JS Cao Q McKenna DH Hippen KL Curtsinger J Infusion of ex vivo expanded T regulatory cells in adults transplanted with umbilical cord blood: safety profile and detection kinetics. Blood (2011) 117:1061–70 10.1182/blood-2010-07-293795 20952687 PMC3035067

[43] Di Ianni M Falzetti F Carotti A Terenzi A Castellino F Bonifacio E Tregs prevent GVHD and promote immune reconstitution in HLA-haploidentical transplantation. Blood (2011) 117:3921–8 10.1182/blood-2010-10-311894 21292771

[44] Tang Q Lee K Regulatory T-cell therapy for transplantation: how many cells do we need? Curr Opin Organ Transplant (2012) 17:349–54 10.1097/MOT.0b013e328355a992 22790069

[45] Hoffmann P Eder R Kunz-Schughart LA Andreesen R Edinger M Large-scale in vitro expansion of polyclonal human CD4(+)CD25high regulatory T cells. Blood (2004) 104:895–903 10.1182/blood-2004-01-0086 15090447

[46] Curotto de Lafaille MA Lafaille JJ Natural and adaptive foxp3+ regulatory T cells: more of the same or a division of labor? Immunity (2009) 30:626–35 10.1016/j.immuni.2009.05.002 19464985

[47] Chen W Jin W Hardegen N Lei K-J Li L Marinos N Conversion of peripheral CD4+CD25- naive T cells to CD4+CD25+ regulatory T cells by TGF-beta induction of transcription factor Foxp3. J Exp Med (2003) 198:1875–86 10.1084/jem.20030152 14676299 PMC2194145

[48] Barrat FJ Cua DJ Boonstra A Richards DF Crain C Savelkoul HF In vitro generation of interleukin 10-producing regulatory CD4(+) T cells is induced by immunosuppressive drugs and inhibited by T helper type 1 (Th1)- and Th2-inducing cytokines. J Exp Med (2002) 195:603–16 10.1084/jem.20011629 11877483 PMC2193760

[49] Chen W Liang X Peterson AJ Munn DH Blazar BR The indoleamine 2,3-dioxygenase pathway is essential for human plasmacytoid dendritic cell-induced adaptive T regulatory cell generation. J Immunol (2008) 181:5396–404 18832696 10.4049/jimmunol.181.8.5396 PMC2614675

[50] Benson MJ Pino-Lagos K Rosemblatt M Noelle RJ All-trans retinoic acid mediates enhanced T reg cell growth, differentiation, and gut homing in the face of high levels of co-stimulation. J Exp Med (2007) 204:1765–74 10.1084/jem.20070719 17620363 PMC2118687

[51] Moon C Kim SH Park KS Choi BK Lee HS Park JB Use of epigenetic modification to induce FOXP3 expression in naïve T cells. Transplant Proc (2009) 41:1848–54 10.1016/j.transproceed.2009.02.101 19545742

[52] Lan Q Fan H Quesniaux V Ryffel B Liu Z Zheng SG Induced Foxp3(+) regulatory T cells: a potential new weapon to treat autoimmune and inflammatory diseases? J Mol Cell Biol (2012) 4:22–8 10.1093/jmcb/mjr039 22107826 PMC3491614

[53] Ohkura N Kitagawa Y Sakaguchi S Development and Maintenance of Regulatory T cells. Immunity (2013) 38:414–23 10.1016/j.immuni.2013.03.002 23521883

[54] Francis RS Feng G Tha-In T Lyons IS Wood KJ Bushell A Induction of transplantation tolerance converts potential effector T cells into graft-protective regulatory T cells. Eur J Immunol (2011) 41:726–38 10.1002/eji.201040509 21243638 PMC3175037

[55] Wood KJ Bushell AR Darby CR Pearson TC West L Morris PJ Mechanism of induction of transplantation tolerance using donor antigen and anti-CD4 monoclonal antibody. Transplant Proc (1991) 23:133–4 1990498

[56] Bushell A Morris PJ Wood KJ Induction of operational tolerance by random blood transfusion combined with anti-CD4 antibody therapy. A protocol with significant clinical potential. Transplantation (1994) 58:133–9 10.1097/00007890-199405820-00002 8042231

[57] Bushell A Morris PJ Wood KJ Transplantation tolerance induced by antigen pretreatment and depleting anti-CD4 antibody depends on CD4+ T cell regulation during the induction phase of the response. Eur J Immunol (1995) 25:2643–9 10.1002/eji.1830250936 7589139

[58] Saitovitch D Bushell A Morris PJ Wood KJ Modulation of the CD4 molecule is a major event in the induction of transplantation tolerance with anti-CD4 monoclonal antibodies. Transplant Proc (1997) 29:1159 10.1016/S0041-1345(96)00504-0 9123250

[59] Hara M Kingsley CI Niimi M Read S Turvey SE Bushell AR IL-10 is required for regulatory T cells to mediate tolerance to alloantigens in vivo. J Immunol (2001) 166:3789–96 11238621 10.4049/jimmunol.166.6.3789

[60] Graca L Thompson S Lin C-Y Adams E Cobbold SP Waldmann H Both CD4(+)CD25(+) and CD4(+)CD25(-) regulatory cells mediate dominant transplantation tolerance. J Immunol (2002) 168:5558–65 12023351 10.4049/jimmunol.168.11.5558

[61] Webster KE Walters S Kohler RE Mrkvan T Boyman O Surh CD In vivo expansion of T reg cells with IL-2-mAb complexes: induction of resistance to EAE and long-term acceptance of isletallografts without immunosuppression. J Exp Med (2009) 206:751–60 10.1084/jem.20082824 19332874 PMC2715127

[62] Adeegbe D Serafini P Bronte V Zoso A Ricordi C Inverardi L In vivo induction of myeloid suppressor cells and CD4(+)Foxp3(+) T regulatory cells prolongs skin allograft survival in mice. Cell Transplant (2011) 20:941–54 10.3727/096368910X540621 21054938

[63] Koreth J Matsuoka K Kim HT McDonough SM Bindra B Alyea EP III Interleukin-2 and regulatory T cells in graft-versus-host disease. N Engl J Med (2011) 365:2055–66 10.1056/NEJMoa1108188 22129252 PMC3727432

[64] Burchill MA Yang J Vogtenhuber C Blazar BR Farrar MA IL-2 receptor beta-dependent STAT5 activation is required for the development of Foxp3+ regulatory T cells. J Immunol (2007) 178:280–90 17182565 10.4049/jimmunol.178.1.280

[65] Delgoffe GM Kole TP Zheng Y Zarek PE Matthews KL Xiao B The mTOR kinase differentially regulates effector and regulatory T cell lineage commitment. Immunity (2009) 30:832–44 10.1016/j.immuni.2009.04.014 19538929 PMC2768135

[66] Zeiser R Leveson-Gower DB Zambricki EA Kambham N Beilhack A Loh J Differential impact of mammalian target of rapamycin inhibition on CD4+CD25+Foxp3+ regulatory T cells compared with conventional CD4+ T cells. Blood (2008) 111:453–62 10.1182/blood-2007-06-094482 17967941 PMC2200823

[67] Battaglia M Stabilini A Roncarolo M-G Rapamycin selectively expands CD4+CD25+FoxP3+ regulatory T cells. Blood (2005) 105:4743–8 10.1182/blood-2004-10-3932 15746082

[68] Zhang P Tey S-K Koyama M Kuns RD Olver SD Lineburg KE Induced regulatory T cells promote tolerance when stabilized by rapamycin and IL-2 in vivo. J Immunol (2013) 191:5291–303 10.4049/jimmunol.1301181 24123683

[69] Hendrikx TK Velthuis JHL Klepper M van Gurp E Geel A Schoordijk W Monotherapy rapamycin allows an increase of CD4 CD25 FoxP3 T cells in renal recipients. Transpl Int (2009) 22:884–91 10.1111/j.1432-2277.2009.00890.x 19453998

[70] Lopez M Clarkson MR Albin M Sayegh MH Najafian N A novel mechanism of action for anti-thymocyte globulin: induction of CD4+CD25+Foxp3+ regulatory T cells. J Am Soc Nephrol (2006) 17:2844–53 10.1681/ASN.2006050422 16914538

[71] D’Addio F Yuan X Habicht A Williams J Ruzek M Iacomini J A novel clinically relevant approach to tip the balance toward regulation in stringent transplant model. Transplantation (2010) 90:260–9 10.1097/TP.0b013e3181e64217 20712076 PMC4140399

[72] Stary G Klein I Bauer W Koszik F Reininger B Kohlhofer S Glucocorticosteroids modify Langerhans cells to produce TGF-beta and expand regulatory T cells. J Immunol (2011) 186:103–12 10.4049/jimmunol.1002485 21135170

[73] Bloom DD Chang Z Fechner JH Dar W Polster SP Pascual J CD4+ CD25+ FOXP3+ regulatory T cells increase de novo in kidney transplant patients after immunodepletion with Campath-1H. Am J Transplant (2008) 8:793–802 10.1111/j.1600-6143.2007.02134.x 18261176

[74] Presser D Sester U Mohrbach J Janssen M Köhler H Sester M Differential kinetics of effector and regulatory T cells in patients on calcineurin inhibitor-based drug regimens. Kidney Int (2009) 76:557–66 10.1038/ki.2009.198 19494797

[75] Brandt C Pavlovic V Radbruch A Worm M Baumgrass R Low-dose cyclosporine A therapy increases the regulatory T cell population in patients with atopic dermatitis. Allergy (2009) 64:1588–96 10.1111/j.1398-9995.2009.02054.x 19432936

[76] Baumgrass R Brandt C Wegner F Abdollahnia M Worm M Low-dose, but not high-dose, cyclosporin A promotes regulatory T-cell induction, expansion, or both. J Allergy Clin Immunol (2010) 126:183–4 10.1016/j.jaci.2010.04.032 20542321

[77] Brandt C Liman P Bendfeldt H Mueller K Reinke P Radbruch A Whole blood flow cytometric measurement of NFATc1 and IL-2 expression to analyze cyclosporine A-mediated effects in T cells. Cytometry A (2010) 77:607–13 10.1002/cyto.a.20928 20583270

[78] Weng J Lai P Lv M Lin S Ling W Geng S Bortezomib modulates regulatory T cell subpopulations in the process of acute graft-versus-host disease. Clin Lab (2013) 59:51–8 23505906 10.7754/clin.lab.2012.120215

[79] Mao R Xiao W Liu H Chen B Yi B Kraj P Systematic evaluation of 640 FDA drugs for their effect on CD4(+)Foxp3(+) regulatory T cells using a novel cell-based high throughput screening assay. Biochem Pharmacol (2013) 85:1513–24 10.1016/j.bcp.2013.03.013 23537702

[80] Zhou X Bailey-Bucktrout SL Jeker LT Penaranda C Martínez-Llordella M Ashby M Instability of the transcription factor Foxp3 leads to the generation of pathogenic memory T cells in vivo. Nat Immunol (2009) 10:1000–7 10.1038/ni.1774 19633673 PMC2729804

[81] Ayyoub M Deknuydt F Raimbaud I Dousset C Leveque L Bioley G Human memory FOXP3+ Tregs secrete IL-17 ex vivo and constitutively express the T(H)17 lineage-specific transcription factor RORgamma t. Proc Natl Acad Sci U S A (2009) 106:8635–40 10.1073/pnas.0900621106 19439651 PMC2688993

[82] Komatsu N Mariotti-Ferrandiz ME Wang Y Malissen B Waldmann H Hori S Heterogeneity of natural Foxp3+ T cells: a committed regulatory T-cell lineage and an uncommitted minor population retaining plasticity. Proc Natl Acad Sci U S A (2009) 106:1903–8 10.1073/pnas.0811556106 19174509 PMC2644136

[83] Voo KS Wang Y-H Santori FR Boggiano C Wang Y-H Arima K Identification of IL-17-producing FOXP3+ regulatory T cells in humans. Proc Natl Acad Sci U S A (2009) 106:4793–8 10.1073/pnas.0900408106 19273860 PMC2653560

[84] Duarte JH Zelenay S Bergman M-L Martins AC Demengeot J Natural Treg cells spontaneously differentiate into pathogenic helper cells in lymphopenic conditions. Eur J Immunol (2009) 39:948–55 10.1002/eji.200839196 19291701

[85] Miyao T Floess S Setoguchi R Luche H Fehling HJ Waldmann H Plasticity of Foxp3(+) T cells reflects promiscuous Foxp3 expression in conventional T cells but not reprogramming of regulatory T cells. Immunity (2012) 36:262–75 10.1016/j.immuni.2011.12.012 22326580

[86] Ohkura N Hamaguchi M Morikawa H Sugimura K Tanaka A Ito Y T cell receptor stimulation-induced epigenetic changes and Foxp3 expression are independent and complementary events required for Treg cell development. Immunity (2012) 37:785–99 10.1016/j.immuni.2012.09.010 23123060

[87] Sharma MD Huang L Choi J-H Lee E-J Wilson JM Lemos H An inherently bifunctional subset of Foxp3+ T helper cells is controlled by the transcription factor eos. Immunity (2013) 38:998–1012 10.1016/j.immuni.2013.01.013 23684987 PMC3681093

[88] Kim H-P Leonard WJ CREB/ATF-dependent T cell receptor-induced FoxP3 gene expression: a role for DNA methylation. J Exp Med (2007) 204:1543–51 10.1084/jem.20070109 17591856 PMC2118651

[89] Tao R de Zoeten EF Ozkaynak E Chen C Wang L Porrett PM Deacetylase inhibition promotes the generation and function of regulatory T cells. Nat Med (2007) 13:1299–307 10.1038/nm1652 17922010

[90] Riley JL June CH The CD28 family: a T-cell rheostat for therapeutic control of T-cell activation. Blood (2005) 105:13–21 10.1182/blood-2004-04-1596 15353480

[91] Suntharalingam G Perry MR Ward S Brett SJ Castello-Cortes A Brunner MD Cytokine storm in a phase 1 trial of the anti-CD28 monoclonal antibody TGN1412. N Engl J Med (2006) 355:1018–28 10.1056/NEJMoa063842 16908486

[92] Lal G Bromberg JS Epigenetic mechanisms of regulation of Foxp3 expression. Blood (2009) 114:3727–35 10.1182/blood-2009-05-219584 19641188 PMC2773485

[93] Lal G Zhang N van der Touw W Ding Y Ju W Bottinger EP Epigenetic regulation of Foxp3 expression in regulatory T cells by DNA methylation. J Immunol (2009) 182:259–73 19109157 10.4049/jimmunol.182.1.259 PMC3731994

[94] Hancock WW Akimova T Beier UH Liu Y Wang L HDAC inhibitor therapy in autoimmunity and transplantation. Ann Rheum Dis (2012) 71(Suppl 2):i46–54 10.1136/annrheumdis-2011-200593 22460138

[95] Ford ML Larsen CP Transplantation tolerance: memories that haunt us. Sci Transl Med (2011) 3:86s22 10.1126/scitranslmed.3002504 21653828

[96] Lee K Nguyen V Lee K-M Kang S-M Tang Q Attenuation of donor-reactive T cells allows effective control of allograft rejection using regulatory T cell therapy. Am J Transplant (2014) 14:27–38 10.1111/ajt.12509 24354870 PMC5262439

[97] Weaver TA Charafeddine AH Agarwal A Turner AP Russell M Leopardi FV Alefacept promotes co-stimulation blockade based allograft survival in nonhuman primates. Nat Med (2009) 15:746–9 10.1038/nm.1993 19584865 PMC2772128

[98] Posselt AM Bellin MD Tavakol M Szot GL Frassetto LA Masharani U Islet transplantation in type 1 diabetics using an immunosuppressive protocol based on the anti-LFA-1 antibody efalizumab. Am J Transplant (2010) 10:1870–80 10.1111/j.1600-6143.2010.03073.x 20659093 PMC2911648

[99] Tavazzi E Ferrante P Khalili K Progressive multifocal leukoencephalopathy: an unexpected complication of modern therapeutic monoclonal antibody therapies. Clin Microbiol Infect (2011) 17:1776–80 10.1111/j.1469-0691.2011.03653.x 22082208 PMC4659503

[100] Levings MK Sangregorio R Roncarolo MG Human cd25(+)cd4(+) T regulatory cells suppress naive and memory T cell proliferation and can be expanded in vitro without loss of function. J Exp Med (2001) 193:1295–302 10.1084/jem.193.11.1295 11390436 PMC2193376

[101] Tang Q Bluestone JA The Foxp3+ regulatory T cell: a jack of all trades, master of regulation. Nat Immunol (2008) 9:239–44 10.1038/ni1572 18285775 PMC3075612

[102] Shevach EM Mechanisms of foxp3+ T regulatory cell-mediated suppression. Immunity (2009) 30:636–45 10.1016/j.immuni.2009.04.010 19464986

[103] Lechler RI Sykes M Thomson AW Turka LA Organ transplantation – how much of the promise has been realized? Nat Med (2005) 11:605–13 10.1038/nm1251 15937473

[104] Schraven B Kalinke U CD28 superagonists: what makes the difference in humans? Immunity (2008) 28:591–5 10.1016/j.immuni.2008.04.003 18482560

[105] Golab K Leveson-Gower D Wang X-J Grzanka J Marek-Trzonkowska N Krzystyniak A Challenges in cryopreservation of regulatory T cells (Tregs) for clinical therapeutic applications. Int Immunopharmacol (2013) 16:371–5 10.1016/j.intimp.2013.02.001 23428908

[106] Peters JH Preijers FW Woestenenk R Hilbrands LB Koenen HJPM Joosten I Clinical grade Treg: GMP isolation, improvement of purity by CD127 depletion, Treg expansion, and Treg cryopreservation. PLoS One (2008) 3:e3161 10.1371/journal.pone.0003161 18776930 PMC2522271

[107] Mougiakakos D Choudhury A Lladser A Kiessling R Johansson CC Regulatory T cells in cancer. Adv Cancer Res (2010) 107:57–117 10.1016/S0065-230X(10)07003-X 20399961

[108] Socié G Blazar BR Acute graft-versus-host disease: from the bench to the bedside. Blood (2009) 114:4327–36 10.1182/blood-2009-06-204669 19713461 PMC2777122

[109] Hoffmann P Ermann J Edinger M Fathman CG Strober S Donor-type CD4(+)CD25(+) regulatory T cells suppress lethal acute graft-versus-host disease after allogeneic bone marrow transplantation. J Exp Med (2002) 196:389–99 10.1084/jem.20020399 12163567 PMC2193938

[110] Edinger M Hoffmann P Regulatory T cells in stem cell transplantation: strategies and first clinical experiences. Curr Opin Immunol (2011) 23:679–84 10.1016/j.coi.2011.06.006 21802270

[111] Issa F Wood KJ Translating tolerogenic therapies to the clinic – where do we stand? Front Immunol (2012) 3:254 10.3389/fimmu.2012.00254 22934094 PMC3422982

[112] Taylor PA Noelle RJ Blazar BR CD4(+)CD25(+) immune regulatory cells are required for induction of tolerance to alloantigen via costimulatory blockade. J Exp Med (2001) 193:1311–8 10.1084/jem.193.11.1311 11390438 PMC2193378

[113] Dodd-o JM Lendermon EA Miller HL Zhong Q John ER Jungraithmayr WM CD154 blockade abrogates allospecific responses and enhances CD4(+) regulatory T-cells in mouse orthotopic lung transplant. Am J Transplant (2011) 11:1815–24 10.1111/j.1600-6143.2011.03623.x 21827610 PMC3827913

[114] Nadig SN Wieckiewicz J Wu DC Warnecke G Zhang W Luo S In vivo prevention of transplant arteriosclerosis by ex vivo-expanded human regulatory T cells. Nat Med (2010) 16:809–13 10.1038/nm.2154 20473306 PMC2929438

[115] Schliesser U Streitz M Sawitzki B Tregs: application for solid-organ transplantation. Curr Opin Organ Transplant (2012) 17:34–41 10.1097/MOT.0b013e32834ee69f 22143395

[116] Soyka MB Holzmann D Akdis CA Regulatory cells in allergen-specific immunotherapy. Immunotherapy (2012) 4:389–96 10.2217/imt.12.10 22512633

[117] Akdis M Verhagen J Taylor A Karamloo F Karagiannidis C Crameri R Immune responses in healthy and allergic individuals are characterized by a fine balance between allergen-specific T regulatory 1 and T helper 2 cells. J Exp Med (2004) 199:1567–75 10.1084/jem.20032058 15173208 PMC2211782

[118] Jin H-S Park Y Elly C Liu Y-C Itch expression by Treg cells controls Th2 inflammatory responses. J Clin Invest (2013) 123:4923–34 10.1172/JCI69355 24135136 PMC3809788

[119] Rolland JM Gardner LM O’Hehir RE Functional regulatory T cells and allergen immunotherapy. Curr Opin Allergy Clin Immunol (2010) 10:559–66 10.1097/ACI.0b013e32833ff2b2 20859202

[120] Zhang Y Zhen H Yao W Bian F Mao X Yang X Antidepressant drug, desipramine, alleviates allergic rhinitis by regulating Treg and Th17 cells. Int J Immunopathol Pharmacol (2013) 26:107–15 23527713 10.1177/039463201302600110

[121] Tritt M Sgouroudis E D’Hennezel E Albanese A Piccirillo CA Functional waning of naturally occurring CD4+ regulatory T-cells contributes to the onset of autoimmune diabetes. Diabetes (2008) 57:113–23 10.2337/db06-1700 17928397

[122] Miyara M Amoura Z Parizot C Badoual C Dorgham K Trad S Global natural regulatory T cell depletion in active systemic lupus erythematosus. J Immunol (2005) 175:8392–400 16339581 10.4049/jimmunol.175.12.8392

[123] Valencia X Stephens G Goldbach-Mansky R Wilson M Shevach EM Lipsky PE TNF downmodulates the function of human CD4+CD25hi T-regulatory cells. Blood (2006) 108:253–61 10.1182/blood-2005-11-4567 16537805 PMC1895836

[124] Viglietta V Baecher-Allan C Weiner HL Hafler DA Loss of functional suppression by CD4+CD25+ regulatory T cells in patients with multiple sclerosis. J Exp Med (2004) 199:971–9 10.1084/jem.20031579 15067033 PMC2211881

[125] Baecher-Allan C Hafler DA Human regulatory T cells and their role in autoimmune disease. Immunol Rev (2006) 212:203–16 10.1111/j.0105-2896.2006.00417.x 16903916

[126] Tarbell KV Petit L Zuo X Toy P Luo X Mqadmi A Dendritic cell-expanded, islet-specific CD4+ CD25+ CD62L+ regulatory T cells restore normoglycemia in diabetic NOD mice. J Exp Med (2007) 204:191–201 10.1084/jem.20061631 17210729 PMC2118426

[127] Sakaguchi S Ono M Setoguchi R Yagi H Hori S Fehervari Z Foxp3+ CD25+ CD4+ natural regulatory T cells in dominant self-tolerance and autoimmune disease. Immunol Rev (2006) 212:8–27 10.1111/j.0105-2896.2006.00427.x 16903903

[128] Marek-Trzonkowska N Mysliwiec M Dobyszuk A Grabowska M Techmanska I Juscinska J Administration of CD4+CD25highCD127- regulatory T cells preserves beta-cell function in type 1 diabetes in children. Diabetes Care (2012) 35:1817–20 10.2337/dc12-0038 22723342 PMC3425004

[129] Souroujon MC Aricha R Feferman T Mizrachi K Reuveni D Fuchs S Regulatory T cell-based immunotherapies in experimental autoimmune myasthenia gravis. Ann N Y Acad Sci (2012) 1274:120–6 10.1111/j.1749-6632.2012.06844.x 23252906

[130] Zhou X Kong N Wang J Fan H Zou H Horwitz D Cutting edge: all-trans retinoic acid sustains the stability and function of natural regulatory T cells in an inflammatory milieu. J Immunol (2010) 185:2675–9 10.4049/jimmunol.1000598 20679534 PMC3098624

[131] Bagavant H Tung KSK Failure of CD25+ T cells from lupus-prone mice to suppress lupus glomerulonephritis and sialadenitis. J Immunol (2005) 175:944–50 16002693 10.4049/jimmunol.175.2.944

[132] Huter EN Stummvoll GH DiPaolo RJ Glass DD Shevach EM Cutting edge: antigen-specific TGF beta-induced regulatory T cells suppress Th17-mediated autoimmune disease. J Immunol (2008) 181:8209–13 19050237 10.4049/jimmunol.181.12.8209 PMC2788513

[133] Pasare C Medzhitov R Toll pathway-dependent blockade of CD4+CD25+ T cell-mediated suppression by dendritic cells. Science (2003) 299:1033–6 10.1126/science.1078231 12532024

[134] D’Alessio FR Tsushima K Aggarwal NR West EE Willett MH Britos MF CD4+CD25+Foxp3+ Tregs resolve experimental lung injury in mice and are present in humans with acute lung injury. J Clin Invest (2009) 119:2898–913 10.1172/JCI36498 19770521 PMC2752062

[135] Garibaldi BT D’Alessio FR Mock JR Files DC Chau E Eto Y Regulatory T cells reduce acute lung injury fibroproliferation by decreasing fibrocyte recruitment. Am J Respir Cell Mol Biol (2013) 48:35–43 10.1165/rcmb.2012-0198OC 23002097 PMC3547087

[136] Gandolfo MT Jang HR Bagnasco SM Ko G-J Agreda P Satpute SR Foxp3+ regulatory T cells participate in repair of ischemic acute kidney injury. Kidney Int (2009) 76:717–29 10.1038/ki.2009.259 19625990

[137] Liesz A Suri-Payer E Veltkamp C Doerr H Sommer C Rivest S Regulatory T cells are key cerebroprotective immunomodulators in acute experimental stroke. Nat Med (2009) 15:192–9 10.1038/nm.1927 19169263

[138] Ito T Hanabuchi S Wang Y-H Park WR Arima K Bover L Two functional subsets of FOXP3+ regulatory T cells in human thymus and periphery. Immunity (2008) 28:870–80 10.1016/j.immuni.2008.03.018 18513999 PMC2709453

[139] Flores-Borja F Jury EC Mauri C Ehrenstein MR Defects in CTLA-4 are associated with abnormal regulatory T cell function in rheumatoid arthritis. Proc Natl Acad Sci U S A (2008) 105:19396–401 10.1073/pnas.0806855105 19036923 PMC2614772

[140] Wei S Kryczek I Zou W Regulatory T-cell compartmentalization and trafficking. Blood (2006) 108:426–31 10.1182/blood-2006-01-0177 16537800 PMC1895488

[141] Levine BL Humeau LM Boyer J MacGregor R-R Rebello T Lu X Gene transfer in humans using a conditionally replicating lentiviral vector. Proc Natl Acad Sci U S A (2006) 103:17372–7 10.1073/pnas.0608138103 17090675 PMC1635018

[142] Mekala DJ Geiger TL Immunotherapy of autoimmune encephalomyelitis with redirected CD4+CD25+ T lymphocytes. Blood (2005) 105:2090–2 10.1182/blood-2004-09-3579 15528313

[143] Elinav E Adam N Waks T Eshhar Z Amelioration of colitis by genetically engineered murine regulatory T cells redirected by antigen-specific chimeric receptor. Gastroenterology (2009) 136:1721–31 10.1053/j.gastro.2009.01.049 19208357

[144] Hori S Haury M Coutinho A Demengeot J Specificity requirements for selection and effector functions of CD25+4+ regulatory T cells in anti-myelin basic protein T cell receptor transgenic mice. Proc Natl Acad Sci U S A (2002) 99:8213–8 10.1073/pnas.122224799 12034883 PMC123047

[145] Varela-Rohena A Molloy PE Dunn SM Li Y Suhoski MM Carroll RG Control of HIV-1 immune escape by CD8 T cells expressing enhanced T-cell receptor. Nat Med (2008) 14:1390–5 10.1038/nm.1779 18997777 PMC3008216

